# Linking data sources for measurement of effective coverage in maternal and newborn health: what do we learn from individual- vs ecological-linking methods?

**DOI:** 10.7189/jogh.08.010601

**Published:** 2018-06

**Authors:** Barbara Willey, Peter Waiswa, Darious Kajjo, Melinda Munos, Joseph Akuze, Elizabeth Allen, Tanya Marchant

**Affiliations:** 1 Faculty of Epidemiology and Population Health, London School of Hygiene and Tropical Medicine, London, UK; 2 Makerere University, College of Health Sciences, School of Public Health, Kampala, Uganda; 3 Department of Public Health Sciences, Karolinska Institutet, Stockholm, Sweden; 4 Department of International Health, Johns Hopkins Bloomberg School of Public Health, Baltimore, Maryland, USA; 5 Faculty of Infectious and Tropical Diseases, London School of Hygiene and Tropical Medicine, London, UK

## Abstract

**Background:**

Improving maternal and newborn health requires improvements in the quality of facility-based care. This is challenging to measure: routine data may be unreliable; respondents in population surveys may be unable to accurately report on quality indicators; and facility assessments lack population level denominators. We explored methods for linking access to skilled birth attendance (SBA) from household surveys to data on provision of care from facility surveys with the aim of estimating population level effective coverage reflecting access to quality care.

**Methods:**

We used data from Mayuge District, Uganda. Data from household surveys on access to SBA were linked to health facility assessment census data on readiness to provide basic emergency obstetric and newborn care (BEmONC) in the same district. One individual- and two ecological-linking methods were applied. All methods used household survey reports on where care at birth was accessed. The individual-linking method linked this to data about facility readiness from the specific facility where each woman delivered. The first ecological-linking approach used a district-wide mean estimate of facility readiness. The second used an estimate of facility readiness adjusted by level of health facility accessed. Absolute differences between estimates derived from the different linking methods were calculated, and agreement examined using Lin’s concordance correlation coefficient.

**Results:**

A total of 1177 women resident in Mayuge reported a birth during 2012-13. Of these, 664 took place in facilities within Mayuge, and were eligible for linking to the census of the district’s 38 facilities. 55% were assisted by a SBA in a facility. Using the individual-linking method, effective coverage of births that took place with an SBA in a facility ready to provide BEmONC was just 10% (95% confidence interval CI 3-17). The absolute difference between the individual- and ecological-level linking method adjusting for facility level was one percentage point (11%), and tests suggested good agreement. The ecological method using the district-wide estimate demonstrated poor agreement.

**Conclusions:**

The proportion of women accessing appropriately equipped facilities for care at birth is far lower than the coverage of facility delivery. To realise the life-saving potential of health services, countries need evidence to inform actions that address gaps in the provision of quality care. Linking household and facility-based information provides a simple but innovative method for estimating quality of care at the population level. These encouraging findings suggest that linking data sets can result in meaningful evidence even when the exact location of care seeking is not known.

Observed increases in uptake of facility-based health care by families in high mortality settings [1] have not been consistently associated with increased survival of mothers and newborns [2-5]. In alignment with the Sustainable Development Goals [6], this has led to commitments to improve the quality of care being delivered and to develop measures of effective coverage that reflect quality, life-saving care, including that for mothers and newborns [7-10]. Moving beyond crude coverage to focus on effective coverage, defined as “*the fraction of potential health gain that is actually delivered to the population through the health system, given its capacity*”, shifts the focus to acknowledge the importance of use and quality of services, in addition to need [11]. This focus on effective coverage has highlighted the quality gap in facility-based care for mothers and newborns in a variety of low and middle country settings [12-14].

The ideal source of data on the quality of facility-based care should be routine facility-based data, however there is considerable overlap between settings with high mortality, suboptimal quality of care, and poor quality of routinely collected facility data [15,16]. As such, data often come from population level household surveys. These are needed to determine population level access to health care, however respondents often cannot report on quality measures, especially for clinical care [17]. Data on quality of care are also drawn from health facility readiness assessments [18]. These can provide estimates of readiness to provide good quality clinical care, but do not provide a population level denominator.

It has been proposed that linking these two data streams could provide a way forward for effective coverage methods, and there is a growing body of work that has aimed to achieve this integration of population and quality measurement [19]. In their recent systematic review, Do and colleagues identified 59 studies that linked household and facility data and reported that researchers had taken one of two linking options. The first (i): an individual-linking approach (linking household data to information from the precise point of care accessed). The second (ii): an ecological-linking approach (linking household data to aggregated facility data, or to facility data summarised at a pre-defined level eg, locality, level of health facility accessed, or level of the health care provider accessed by households).

To advance the measurement agenda on linking for effective coverage it is important to understand the relative benefits of individual- and ecological-linking methods. We suggest that individual level linking may provide a gold standard for effective coverage measures as this links the participant’s information from household surveys to the precise health facility at which they sought care. However, the method is very resource intensive and requires that studies be designed purposively to visit every health care outlet accessed by households. This is not likely to be transferrable to large-scale measurement. Ecological level linking is more feasible, including by accessing independent data sources (for example Demographic and Health Surveys and Service Provision Assessments), but may produce less precise estimates of effective coverage measures than individual level linking because the individual household data are linked to an average of the facility unit being linked to and facilities are likely to vary in the quality of care they provide [20].

Between 2011 and 2014 the maternal and newborn health project “EQUIP” (Expanded Quality Management Using Information Power) [21] conducted continuous district-level household surveys in Mayuge district, eastern Uganda alongside a repeat census of health facilities in the same district for the same time period [22]. These data provide us an opportunity to carry out a head-to-head comparison of ecological- and individual-linking methods. Using these data, we demonstrate effective coverage outcomes obtained after application of each linking method, using the individual-linking method as the standard against which we aim to understand the equivalence of the more feasible ecological-linking method.

## METHODS

### Study area

The EQUIP study was a non-randomised quality improvement intervention implemented in one district of eastern Uganda (Mayuge) [23]. Quality improvement is a strategy to improve implementation levels for evidence-based essential interventions. In the EQUIP study collaborative quality improvement teams tested self-identified strategies to support the implementation of essential maternal and newborn interventions recommended by the WHO. Throughout the study, the teams had access to locally-generated high quality health data from a continuous household survey, repeat health facility censuses, complemented by routine data from health facilities.

**Figure 1:**
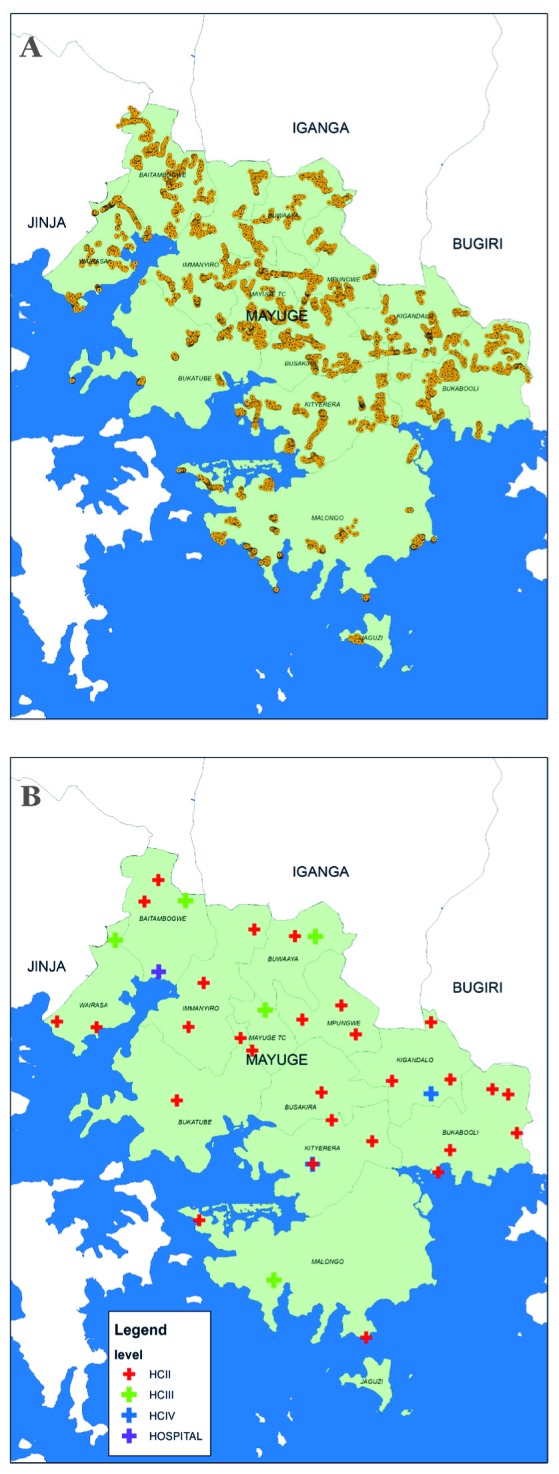
Map of Mayuge district showing location of household clusters and health facilities included in this analysis. (A) Household clusters. Yellow dot – household cluster included in household survey. (B) Health facilities. Red cross – health centre II, Green cross – health centre III, Blue cross – health centre IV, Purple cross – hospital.

Mayuge district has a population of approximately 400 000 people, is predominantly rural, and has an estimated maternal mortality ratio of 438 per 100 000 live births and an estimated neonatal mortality rate of 23 per 1000 livebirths, based on data from the 2011 DHS [24]. In 2014 there were 38 government owned health facilities in the district and no private birthing facilities (**Figure 1**). At the time of the EQUIP study all facilities were conducting births, but level II facilities had only recently been upgraded to conduct births due to increases in demand for facility-based delivery in the locality.

### Data sources

**Figure 2:**
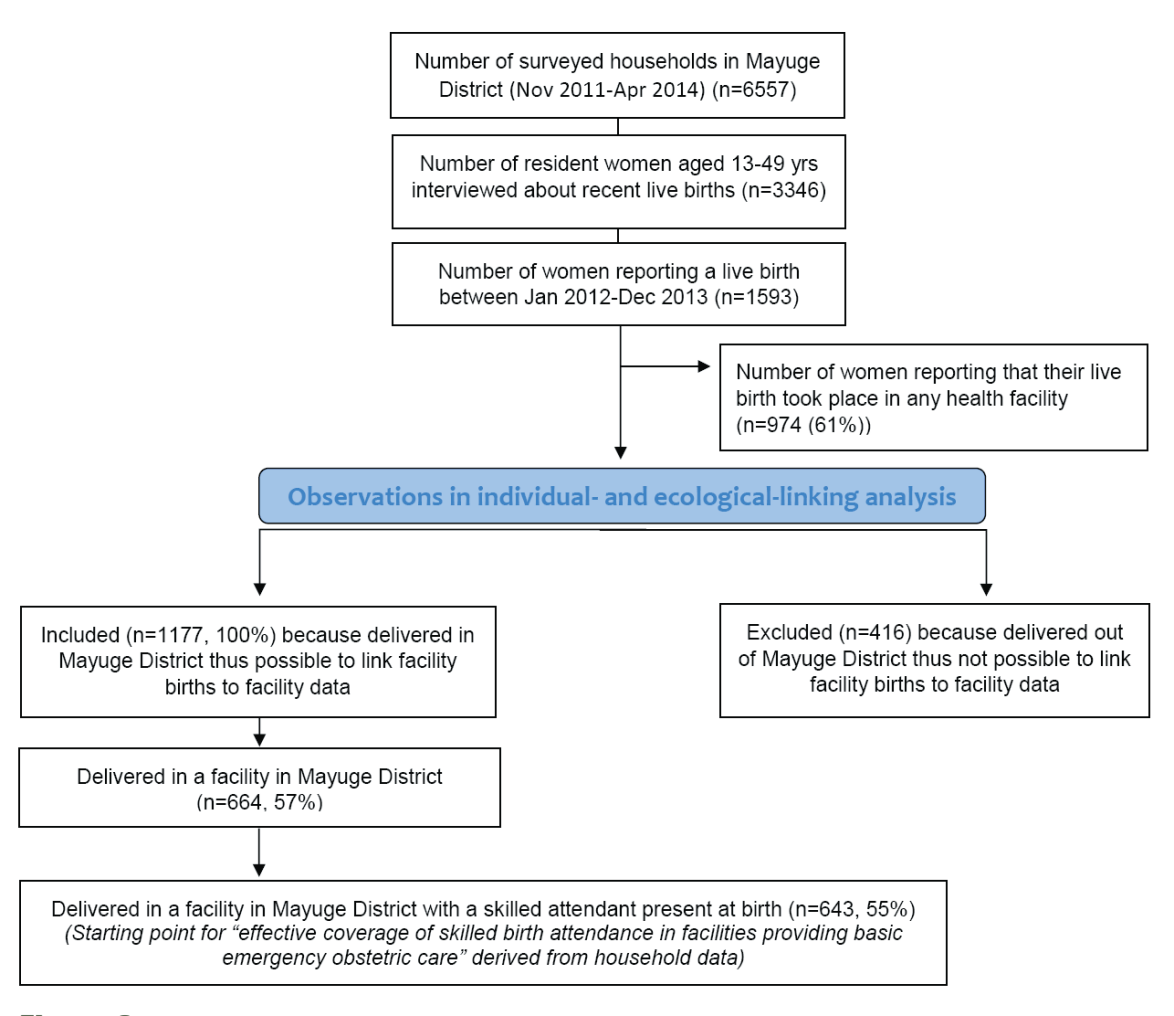
Sample selection.

Full details of the data collection protocol for the project have been reported elsewhere [22]. In brief, a continuous population level cluster survey designed to represent the district at multiple time points, and six repeat health facility censuses in the district were implemented between November 2011 and April 2014 (**Figure 2**). Questionnaires were adapted from Demographic and Health Surveys (DHS) tools [25]. The household survey comprised of a household module capturing information on household characteristics and residents, and a women’s module addressed to all female residents aged 15-49 years. Women aged 15-49 years who reported a live birth in the two years prior to survey were also asked a detailed set of questions about the antenatal, intrapartum, and postnatal care they and their infant had received. The repeat facility census included a modular check-list type questionnaire including staff employed, drugs, supplies and equipment.

For this analysis a sub-set of EQUIP data were analysed. From the continuous household survey data sets inclusion criteria were as follows: women aged 15-49 years who reported a live birth in fixed reference period (24-month period 1 January 2012 and 31 December 2013); and women who delivered in the district of residence (to maximise the potential to link household data on place of birth to facility readiness data) (**Figure 2**). The reported name and level of facility (Health Centre II, III or IV, and hospital) for each included birth were identified and cross-checked against the facility census list. Where inconclusive, reported facility names and levels were returned to the survey field team for final verification.

In total six facility census data sets were available from the EQUIP study. For this analysis, the EQUIP health facility census round three (1 November 2012 – 28 February 2013) was selected as that representing measures of the service environment at the mid-point of the household survey reference period. Variability of quality of care indicators across the six censuses was examined to consider the stability of the quality estimates over time.

### Indicators

Currently, there are not standardised agreed metrics for measuring the of quality of care for maternal and newborn health [8]. In this study we adapted measures from signal functions to provide routine and emergency obstetric and newborn care proposed by Gabrysch and colleagues [18]. Gabrysch and colleagues suggest four dimensions of facility care including general requirements, routine care, basic and comprehensive emergency care. Signal functions refer to health workforce availability and skills as well as availability of commodities to deliver care, in addition to life-saving behaviours [18].

The measure used in our study included the availability of commodities to provide routine care and basic emergency obstetric and newborn care (BEmONC). These were categorised into a binary indicator as to whether the facility had all of these commodities available or not. These components are listed in **Box 1**. These included availability of: infrastructure (electricity and running water); infection prevention measures; commodities to monitor and manage labour; essential medicines; commodities to provide clean cord care; and commodities to carry out neonatal resuscitation (**Box 1**). These six components were combined using equal weighting to represent one binary indicator of ‘facility readiness to provide basic emergency obstetric and newborn care’. These indicators were then linked to household observations on place of birth to estimate effective coverage of skilled birth attendance in a health facility ready to provide BEmONC.

Box 1 **Facility readiness components for indicators representing “facility readiness to provide basic emergency obstetric and newborn care (BEmONC)” based on availability on the day of the survey**

Facility readiness components include:

Infrastructure – had a source of electricity and running water 24hr/day

Infection prevention – had commodities for infection prevention available (disinfectant, disposable gloves, soap, sharps box, sterilizer)

Monitoring labour – had commodities to monitor and manage labour available (blood pressure cuff, timer, urine protein dipstick, fetal stethoscope, thermometer)

Essential drug – had essential drugs for management of complications in mothers and babies available (parenteral antibiotics for maternal infection and newborn sepsis, parenteral anticonvulsants, parenteral oxytocics for haemorrhage and uterotonics for active management of the third stage of labour, AMSTL)

Neonatal resuscitation – had commodities for neonatal resuscitation available (bag and mask)

Clean cord care – had commodities for hygienic core care available (sterile cord cutter and cord tie)

All components – had all commodities for all six indicators available

The household data set included a variable on skilled birth attendance (SBA), constructed using standard definitions. Each of the recent births attended by an SBA was assigned a facility readiness score based on three link methods as described below.

#### Linking method (A): Individual-linking

The individual-linking method was considered as the gold standard as this linked the participant’s information from household surveys to the precise health facility at which they sought care. For individual household observations reporting skilled birth attendance, the facility readiness to provide BEmONC (**Box 1**) was merged in by matching name of health facility between household and facility data sets. Home births were coded as having no facility readiness. Population level tabulations of effective coverage were then made.

#### Linking method (B): Ecological-linking (I)

Ecological-linking was carried out for a mean facility readiness score at the district level – thus not accounting for the number of service users (volume of births) or readiness at different levels in the health system. Using the same household and facility data sets as method A, each facility birth from the household data set was assigned the mean facility readiness status for the district as a whole (all health facilities combined); home births were again coded as having no facility readiness, and population level tabulations of effective coverage were made.

#### Linking method (C): Ecological-linking (II)

In the second ecological-linking method, linking was carried out by level of facility because different levels in the health system were not equally well-equipped and had different numbers of service users. The same household survey sample of women was included as in the individual-linking method. The facility data set was collapsed by level of facility and readiness indicators tabulated for each level (level II, III, IV or hospital). For each individual household observation with a delivery attended by an SBA, the facility readiness status for the reported level of facility was merged in. Home births again were coded as having no facility readiness, and population level tabulations of effective coverage made.

### Analysis of data

For each of the three linked data sets, “effective coverage of skilled birth attendance in facilities providing basic emergency obstetric and newborn care” was calculated as the product of (i) the prevalence of attendance by an SBA in a health facility within Mayuge District and (ii) the prevalence of facility readiness for each quality of care indicator. Confidence intervals surrounding the estimate of effective coverage for each quality of care indicator were calculated using the Delta method [26].

The absolute differences between effective coverage estimates from the three linking methods were examined. Agreement between linking methods was examined using Lin’s concordance correlation coefficient and Bland and Altman plots to investigate the existence of any systematic difference between the measurements (ie, fixed bias) and to identify possible outliers [27].

### Ethics

Ethical clearance for the EQUIP study was obtained from the Uganda National Council of Science and Technology, Makerere University School of Public Health, and the London School of Hygiene and Tropical Medicine (LSHTM). This study underwent human subjects review process at CDC, Atlanta and was approved as not being engaged in human subjects’ research. Advocacy and sensitization meetings with district and sub-district authorities were held at the start of the EQUIP study. Communities and health facilities were informed about the survey by a survey team member one day prior to interview, using information sheets in the local languages. Written, informed consent to participate in the surveys was obtained from household heads, women, facility in-charge, and health staff interviewed. In the case of illiterate participants, the translated informed consent sheet was read aloud to the participant in the presence of a literate neighbourhood witness who confirmed the content of the consent sheet, and informed consent was obtained by means of thumb print from the illiterate participant and signature from the literate neighbourhood witness.

## RESULTS

### Household survey

Throughout the duration of the EQUIP study (November 2011 to April 2014), 6557 households were visited by the continuous household survey team. A total of 3346 resident women aged 15-49 years were listed and interviewed. In total 1593 reported a live birth during the period January 2012 to December 2013, 74% (n = 1177) in their district of residence. Of these, 643 (55%) were attended by a skilled birth attendant in a health facility (**Figure 2**). The volume of births taking place at different facility levels was not evenly distributed. Of the 643 births taking place with a skilled birth attendant in a facility, 39% were in Health Centre II, 32% in Health Centre III, 19% in Health Centre IV and 9% in the district’s one hospital (**Table 1**).

**Table 1:** Household survey reported births, Mayuge district, Uganda, January 2012 – December 2013

Level of health facility	Live births (N)	%	% of facility deliveries (95% CI)
Health Centre II with a skilled birth attendant	251	21	39 (31-48)
Health Centre III with a skilled birth attendant	206	18	32 (25-41)
Health Centre IV with a skilled birth attendant	125	11	19 (11-31)
Hospital with a skilled birth attendant	61	5	9 (6-14)
sub-total	643	55	100
Any health facility with an unskilled birth attendant	21	2	
Home births	513	43	
Total reported births	1177	100	

CI – confidence interval

### Facility indicators

In total, 38 health facilities were included in the facility census, but three Health Centre level II facilities had no linked household report and were excluded from this analysis. Of the remaining 35 facilities, 26 were Health Centre II, six Health Centre III, two Health Centre IV and one hospital (**Table 2**).

**Table 2:** Health facility readiness measures for mid-point facility census, completed during period November 2012 – February 2013 (for linking to household data on births occurring 1 January 2012 – 31 December 2013), showing outcomes for all facilities used in linking analysis (N = 35)

	District wide	By facility level			
	**All facilities**	**Health Centre II**	**Health Centre III**	**Health Centre IV**	**Hospital**
N health facilities named in household survey as location of at least one birth in the previous 24 mo (included for linking)*	35	26	6	2	1
Readiness on the day of survey:	%	%	%	%	%
Infrastructure	29	15	67	50	100
Infection prevention	60	53	83	50	100
Monitoring labour	14	0	50	50	100
Essential drugs	26	8	67	100	100
Neonatal resuscitation	37	19	83	100	100
Clean cord care	49	34	100	100	100
All components	9	0	33	0	100

*In total, 38 health facilities were included in the facility census, but 3 (health centre level II) were not a delivery facility used by any of the household respondents, thus excluded from this analysis.

The six facility readiness indicators tabulated for these 35 health facilities by level of facility are shown in **Table 2**. Large differences were observed in readiness both between different readiness indicators and within readiness indicators by level of facility. As expected, all indicators were present at the hospital. Health Centre III and IV had relatively high readiness outcomes for all indicators, lowest being infrastructure (67% and 50% of these facilities respectively) and availability of commodities for monitoring labour (50% of facilities).

Health Centre level II – representing the largest number of facilities in the district (n = 26) and 39% of included livebirths from the household survey had very low readiness outcomes for all indicators. Only 8% of these Health Centre II facilities had essential medicines in stock on the day of the survey, 19% had newborn bag and masks available for neonatal resuscitation, and none had all the required commodities available to manage and monitor labour (principally due to the lack of blank partographs).

To examine the stability of our readiness measures over time we tabulated the availability of commodities to provide basic emergency obstetric and newborn care for all six available health facility censuses. Of our six components, two (infrastructure and availability of essential drugs) were stable over time. The remaining four components (**Box 1**) showed some variability between censuses, although this was within two standard deviations of the mean prevalence for all six censuses (data not shown).

### Effective coverage

Taking individual-level linking as our gold standard we observed that during 2012-13, the population level effective coverage of births in Mayuge district that took place with a skilled birth attendant in a facility ready to provide basic emergency obstetric care was just 10% (95% CI 3-17) (**Table 3**).

**Table 3:** Effective coverage of skilled birth attendance in a facility ready to provide BEmONC, Mayuge district, using individual- and ecological-linking methods*

	A† Individual linking method: (gold standard)	B^‡^ Ecological-linking method (1): (no adjustment for facility level)	A–B Absolute difference (%)	C§ Ecological-linking method (2): (adjusted for facility level)	A–C Absolute difference (%)
Effective coverage of births with a skilled attendant in a health facility ready to provide:	% (95% CI)	% (95% CI)		% (95% CI)	
Infrastructure	31.09 (25.08, 37.11)	15.61 (7.20, 24.02)	+15.48	25.44 (7.71, 43.17)	+5.65
Infection prevention	37.29 (31.46, 43.14)	32.78 (23.34, 42.21)	+ 4.51	36.56 (18.91, 54.21)	–0.73
Monitoring labour	16.48 (11.66, 21.30)	7.80 (1.34, 14.27)	+8.68	19.24 (4.16, 34.33)	–2.76
Essential drugs	26.42 (20.41, 32.44)	14.05 (5.64, 22.46)	+12.37	29.11 (11.38, 46.84)	–2.69
Neonatal resuscitation	30.08 (25.17, 34.98)	20.29 (11.24, 29.34)	+9.79	34.49 (24.38, 44.60)	–4.41
Clean cord care	39.08 (32.44, 45.72)	26.53 (17.07, 36.00)	+12.55	35.38 (21.92, 48.83)	+3.70
All components	9.86 (3.21, 16.50)	4.68 (21.37, 31.69)	+5.18	11.02 (3.85, 18.19)	–1.16

CI – confidence interval

*Total number of births as shown in **Figure 2**: N = 1177; of these, number that occurred in a health facility with a skilled attendant present (linked for effective coverage): n = 643 (55%).

†Direct individual-linking between named facility of birth or home birth (**Table 1**, household) and readiness status in that exact facility (**Table 2**, facility).

‡Applying distribution of facility or home births (**Table 1**, household) to facility readiness for all facilities in the district combined (**Table 2**, facility).

§Applying distribution of facility level of birth or home births (**Table 1**, household) to facility readiness by level of facility (**Table 2**, facility).

### Comparison of linking methods

**Figure 3:**
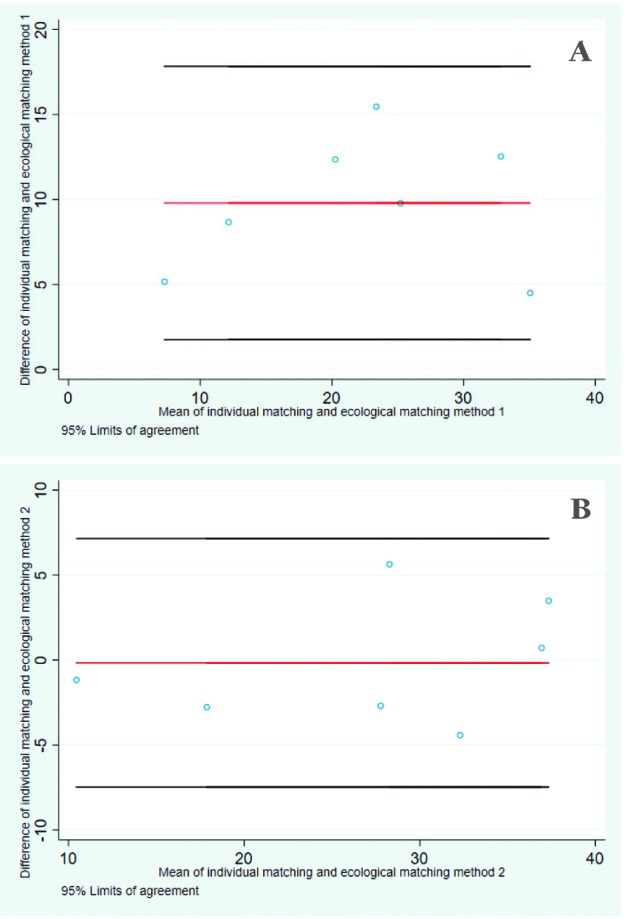
Bland and Altman plots with 95% limits of agreement comparing individual- and ecological-linking methods. (A) Ecological-linking (no adjustment for facility level). (B) Ecological-linking (adjusted for facility level).

Differences between this gold standard effective coverage method and the ecological level linking method (ii) (that adjusted for facility level) were small (**Table 3**). For all signal functions combined the absolute difference was within one percentage point. Across the range of individual facility readiness for BEmONC indicators the absolute difference was within plus or minus six percentage points. For agreement, Lin’s concordance correlation coefficient was 0.92 (0.63-0.98), supporting the use of this ecological-linking method as a proxy for individual-linking. This was also borne out by the Bland and Altman plots which showed no fixed bias (mean difference: –0.29 (–3.83 - 3.26)) and no evidence of any outliers (**Figure 3**). The 95% limits of agreement of -7 to 7 suggest that the two linking approaches are unlikely to differ by more than that for most indicators.

However, differences between the gold standard and ecological-linking method (i) (that made no adjustment for facility level) were larger (**Table 3**). For all facility readiness for BEmONC indicators combined the absolute difference was five percentage points lower than the individual-linking. For each indicator separately this linking method consistently resulted in lower estimates, with absolute percentage point differences between five and 15 percentage points lower than the individual-linking method (**Table 3**). Less agreement was observed between this ecological-linking method and the individual-linking. Lin’s concordance correlation coefficient was 0.57 (0.13-0.82), demonstrating a lack of agreement. The Bland and Altman plots also demonstrated fixed bias with a mean difference of 10.18 (6.46-13.90) (**Figure 3**).

## DISCUSSION

Effective coverage measures that adjust crude population level coverage for readiness to provide quality care may provide a powerful mechanism for revealing gaps in the delivery of effective, life-saving care. Indeed, data from Uganda’s most recent DHS (2016) show that despite dramatic increases from 2011 levels in prevalence of facility delivery (from 57% to 74%) and skilled birth attendance (from 58% to 73%), and substantial declines in under-five and infant mortality rates, the neonatal mortality rate (heavily influenced by deaths within the first 24 hours after delivery) has failed to show a corresponding decline over the past 15 years [28].

In this analysis, using gold-standard individually-linked household and facility data on place of birth we observed that the coverage of skilled birth attendance in a health facility in Mayuge district, Uganda was just 10% after building in a measure of health facility readiness to provide basic emergency obstetric care, in comparison to the crude coverage of 55%. This aggregation of input data provided a useful take away message but was made more actionable for governments by breaking down the readiness measure, allowing quality improvement initiatives to take targeted action to improve outcomes.

Substantial overestimates in the life-saving potential of crude coverage compared to effective coverage estimates of life-saving interventions for mothers and newborns have been shown previously, for example in data from Tanzania [12], from India, Ethiopia and Nigeria [13], as well as from DHS-based data from Rwanda, Uganda, Namibia, Kenya, Ethiopia, Ghana and Mozambique [14].

Effective coverage measures that link population level data on access to health care with facility level data on health care quality are increasingly frequently reported [29-31]. However, there is little guidance on appropriate methods for linking: for example, which data to link, for which units, and with what temporal alignment. Consequently, it is likely that the methods for calculating effective coverage differ between reports. Further, the linking of data sets is usually not planned at the outset of data collection meaning that analysts must be opportunistic in the data that they link. Without evidence on the biases associated with different linking methods the potential for interpretation, like-for-like comparisons, and broad-based buy-in of results may be limited.

With access to a relatively large household data set on care at birth, alongside a detailed and temporally-aligned health facility readiness census, we had the opportunity to examine three different approaches to linking data for effective coverage measures on the topic of care at birth. We observed that different levels of health facility were not equally ready, and do not care for equivalent volumes of birth events. The relevance of these two points was borne out by our finding that, compared to the gold standard individual-linking method, linking with adjustment for the level of facility accessed resulted in high agreement and low bias, while linking without adjusting for level of facility resulted in high bias. Failure to take into account this variability underestimated the effective coverage of skilled birth attendance in comparison to the individual-linking estimate. This was because, although Health Centre II units represented 74% of facilities in this district of Uganda, and were less well equipped than other facilities, only 39% of facility deliveries with a skilled birth attendant took place in these units. This need to adjust for variability within the health system may differ by setting. We would encourage exploration of the source of variability (for example health centre level as in our study, but also divisions of public/private, urban/rural, hard to reach or volatile regions), in order to determine the appropriate characteristic for stratification in different contexts.

These findings are encouraging because they suggest that linking data sets can result in meaningful evidence even when the exact location of care seeking is not known. Outside the context of research studies it is very rare to have a census of health care providers but this analysis suggests that survey data disaggregated by facility level may be sufficiently meaningful, at least until reliable routine facility data for linking becomes the norm.

### Limitations

Our data were drawn from one district in eastern Uganda, based on a representative household survey and a health facility census. Household surveys themselves are susceptible to measurement error, for example respondents may incorrectly report the cadre of health worker, but these errors are present for crude as well as effective coverage measurement. Facility readiness surveys represent availability on the day of survey and our analysis revealed that some but not all commodities were stable over time.

Importantly, the linking methods agenda needs to be extended and our study has revealed three methodological areas that we highlight for further attention. First, our facility data was unusual in that it was a census of all facilities in the district, not a sample. It will be important to carry out methodological work that explores the effect on the sensitivity of ecological-linking when using large samples of health facilities. Second, quality is multi-dimensional and can include elements across the inputs, process and outcome chain [32,33]. However, our working definition of facility readiness to deliver basic emergency obstetric and newborn care focused on commodities, and did not incorporate availability, training or capability of health facility staff attending births and caring for newborns, nor estimates of coverage of actual life-saving behaviours. As such, the effective coverage estimates calculated speak to capacity to deliver quality care, rather than the quality of care delivered in practice. The inclusion of health-worker process data would add further complexity, for example to account for different cadres of worker within the same facility. However, an advantage of this commodity-based focus is the relative stability of components over time, while staff availability and training or capability are likely to be much less stable due to staff turnover and absence.

Finally, the context of this study was an exclusively public health facility setting: no private providers for care at birth were present in the study area. Methodological studies on linking that represent different health topics, more complex constructs of quality, and different health system complexities would add value to the evidence presented here and in combination may lead to standardised guidance for assessing the strength of effective coverage evidence.

## CONCLUSIONS

To our knowledge, this is the first study to compare individual -and ecological -linking methods to estimate effective coverage in this head-to-head manner in the same population. As with numerous previous studies, we noted a substantial overestimate in coverage of skilled birth attendance in a health facility (55%) in comparison to an estimate of effective coverage of the percent of women delivering in facilities with skilled attendants in facilities where essential commodities are available to provide emergency obstetric and newborn care (10%). Our experience has highlighted that it is methodologically important to account for variability in both volume of births and variability of quality of care at the facility level in the construction of effective coverage measures at the time of birth. In this study this variability was summarised by stratifying by health centre level. Failure to take into account this variability underestimated the effective coverage of skilled birth attendance in comparison to the individual-linking estimate. Along with an extension of linking methods to incorporate more comprehensive measures of readiness to deliver quality care for mothers, newborn and children, we suggest that linking household survey data to appropriately-timed, health facility level-specific ecological data from facility surveys approximates well to estimates obtained by an individually-linked approach, and is a pragmatic approach to estimating effective coverage in settings where routine HMIS data are unreliable.
